# External validation of stroke mimic prediction scales in the emergency department

**DOI:** 10.1186/s12883-020-01846-6

**Published:** 2020-07-07

**Authors:** Tian Ming Tu, Guan Zhong Tan, Seyed Ehsan Saffari, Chee Keong Wee, David Jeremiah Ming Siang Chee, Camlyn Tan, Hoon Chin Lim

**Affiliations:** 1 Department of Neurology, National Neuroscience Institute, Singapore, Singapore; 2 Singhealth Duke-NUS Neuroscience Academic Clinical Program, Singapore, Singapore; 3 Lee Kong Chian School of Medicine, Nanyang Technological University of Singapore, Singapore, Singapore; 4 Centre of Quantitative Medicine, Office of Research, Duke-NUS Medical School, Singapore, Singapore; 5 Clinical Trials and Research Unit, National Neuroscience Institute, Singapore, Singapore; 6 Accident and Emergency Department, Changi General Hospital, Singapore, Singapore

**Keywords:** Thrombolytic therapy, Clinical decision-making, Predictive value of tests, Humans, Adults, Stroke/etiology, Tissue plasminogen activator

## Abstract

**Background:**

Acute ischemic stroke is a time-sensitive emergency where accurate diagnosis is required promptly. Due to time pressures, stroke mimics who present with similar signs and symptoms as acute ischemic stroke, pose a diagnostic challenge to the emergency physician. With limited access to investigative tools, clinical prediction, tools based only on clinical features, may be useful to identify stroke mimics. We aim to externally validate the performance of 4 stroke mimic prediction scales, and derive a novel decision tree, to improve identification of stroke mimics.

**Methods:**

We performed a retrospective cross-sectional study at a primary stroke centre, served by a telestroke hub. We included consecutive patients who were administered intravenous thrombolysis for suspected acute ischemic stroke from January 2015 to October 2017. Four stroke mimic prediction tools (FABS, simplified FABS, Telestroke Mimic Score and Khan Score) were rated simultaneously, using only clinical information prior to administration of thrombolysis. The final diagnosis was ascertained by an independent stroke neurologist. Area under receiver operating curve (AUROC) analysis was performed. A classification tree analysis was also conducted using variables which were found to be significant in the univariate analysis.

**Results:**

Telestroke Mimic Score had the highest discrimination for stroke mimics among the 4 scores tested (AUROC = 0.75, 95% CI = 0.63–0.87). However, all 4 scores performed similarly (DeLong *p* > 0.05). Telestroke Mimic Score had the highest sensitivity (91.3%), while Khan score had the highest specificity (88.2%). All 4 scores had high positive predictive value (88.1 to 97.5%) and low negative predictive values (4.7 to 32.3%). A novel decision tree, using only age, presence of migraine and psychiatric history, had a higher prediction performance (AUROC = 0.80).

**Conclusion:**

Four tested stroke mimic prediction scales performed similarly to identify stroke mimics in the emergency setting. A novel decision tree may improve the identification of stroke mimics.

## Background

Intravenous thrombolysis (IVT) is currently the standard of care for acute ischemic stroke patients presenting within 4.5 h of symptom onset [1]. However, approximately 30% of patients presenting to the Emergency Department with stroke-like symptoms are unfortunately stroke mimics [2, 3], posing a significant diagnostic challenge. Accurate diagnosis of ischemic stroke is critical, as IVT in stroke mimics may result in a small, but significant, life- threatening risk of intracerebral haemorrhage, without any benefit [4, 5].

Despite the availability of a multitude of radiological investigations to exclude stroke mimics, the diagnosis of stroke remains a clinical challenge. Emergent use of non-contrast computed tomography (NCCT) head in patients with stroke-like symptoms is primarily to exclude intracerebral hemorrhage [1] and not to confirm the presence of an ischemic stroke. Although NCCT head may identify early subtle signs of ischemic stroke, it is normal in majority of patients who present within the IVT time-window [6], and does not help to radiologically confirm the diagnosis of ischemic stroke. Other imaging modalities, such as magnetic resonance imaging (MRI) can improve the diagnostic accuracy of ischemic stroke [7, 8], but these may not be universally available in the emergent setting and its routine use is not cost-effective [1].

Stroke mimic prediction scales, based solely on history and examination features, may help to identify stroke mimics in the emergency department. The scales may serve to improve the identification of stroke mimics during decision making for intravenous thrombolysis, in the presence of an emergency physician, a neurologist, and a normal NCCT. Four stroke mimic prediction scores (Table 1) relying only on clinical features have been identified from the literature; namely the FABS [9], simplified FABS (sFABS) [10], the TeleStroke Mimic Score (TMS) [11] and Khan score [12]. These scales may be readily utilised in any emergency setting without relying on advanced radiological investigations or specialty expertise. Using these prediction scales may potentially avert unnecessary IVT and its associated risks in stroke mimic patients who present with stroke-like symptoms.

**Table 1:** Components of Individual Stroke Mimic Prediction Scales

Score	Clinical Variables	Scores	Indicator
FABS	1) Absence of Facial Droop 2) Age < 50 years 3) Absence of Atrial Fibrillation 4) Systolic blood pressure < 150 mmHg at presentation 5) Presence of Isolated sensory deficit 6) History of seizure disorder	1 point per variable. Minimum 0, Maximum 6	Higher the score, **more likely** a stroke mimic
Simplified FABS	1) Absence of Facial Droop 2) Age less than 50 years 3) Absence of Atrial Fibrillation 4) Systolic blood pressure less than 150 mmHg at presentation	1 point per variable. Minimum 0, Maximum 4	Higher the score, **more likely** a stroke mimic
TeleStroke Mimic	1) Age (per year) 2) Atrial Fibrillation 3) Hypertension 4) Seizures 5) Facial weakness 6) NIHSS > 14	+ 0.2 per year + 6 + 3 − 6 + 9 + 5 Minimum: − 6, maximum: no upper limit	Higher the score, **less likely** the stroke mimic
Khan score	1) Age		Higher the score, **more likely** a stroke mimic
	a. < 50	2	
	b. 50–70	1	
	c. > 70	0	
	2) Presence of hypertension/hyperlipidemia/diabetes mellitus/atrial fibrillation (AF):		
	a. None	3	
	b. 1 without AF	2	
	c. 2 or 3 without AF	1	
	d. AF	0	
	3) History of migraine	2	
	4) History of epilepsy	1	
	5) History of psychiatric illness	1	
		Minimum: 0 Maximum: 9	

AF indicates atrial fibrillation; *NIHSS* National Institute of Health Stroke Scale

This study primarily aims to externally validate and compare the predictive performance of the above 4 stroke mimic prediction scores in a single ischemic stroke cohort. We also secondarily aim to determine which of the stroke mimic prediction scores would have best averted thrombolysis in the stroke mimics.

## Methods

We retrospectively reviewed consecutive adult patients (21 years or older) who were administered IVT at a single primary stroke centre (Changi General Hospital, Singapore) between January 2015 and October 2017. All patients administered with IVT in the hospital are captured via a hospital IVT audit registry. This IVT audit registry was used to identify subjects included in this study. All patient management was in accordance with institutional guidelines.

Our stroke centre is a spoke hospital served by a telestroke hub. All stroke patients were assessed together by an on-site emergency physician, and an off-site neurologist, physically located at the hub, over a telestroke system (Krixi Corporation, Georgia, USA). The hub neurologist had access to all radiological investigations in real-time and was able to visualise the patient throughout the telestroke consult. Clinical records of the consult were entered into our hospital emergency department electronic documentation system by the emergency physician, while the neurologist entered the assessment into the telestroke documentation database.

All 4 stroke mimic prediction scales (Table 1) were simultaneously rated by a single independent reviewer, using clinical information electronically recorded by the emergency physician and the neurologist at the time of consult. Only clinical information available prior to IVT administration was used. The rater was blinded to the final diagnosis of ischemic stroke or stroke mimic. The clinical variables collected were age, sex, initial systolic and diastolic blood pressure recordings in the emergency department, and National Institute of Health Stroke Scale (NIHSS) score. Clinical history collected included presence of migraine, history of psychiatric disorder, hypertension, presence of seizures, known diabetes mellitus, and atrial fibrillation. The final diagnosis of ischemic stroke, transient ischemic attack or stroke mimic was determined independently by a second stroke neurologist, who was not part of the study, during the hospitalization. The diagnosis of ischemic stroke was based on presence of an acute infarct detected on neuroimaging (NCCT or MRI) at a later timepoint during the course of hospitalisation. The diagnosis of transient ischemic attack was based on complete resolution of the neurological symptoms within 24 h of symptoms onset, and absence of restriction of diffusion weighted imaging on follow up magnetic resonance imaging at 24 h post-thrombolysis [13]. The diagnosis of stroke mimics was made when patients continued to have persistent symptoms beyond 24 h of symptoms onset, but had no restriction of diffusion weighted imaging on MRI, and hence were not considered to be transient ischemic attacks. This final diagnosis was recorded by a separate study team member, who was not involved in the rating of the clinical variables. We also reviewed the hemorrhagic complication rate of the stroke mimics.

Demographic variables and clinical features were reported as median (range) and frequency (percent) and compared between true stroke and stroke mimic groups using Mann-Whitney U test and Chi-square test for continuous and categorical variables, respectively. The association between these parameters and the four stroke mimic prediction scores with the stroke outcome (stroke mimic versus true stroke) was investigated using univariate logistic regression analysis. Receiver operating characteristic (ROC) analysis was performed to determine the cut-off points for each of the four stroke mimic prediction scores using Youden index, and the sensitivity, specificity, negative predictive value (NPV) and positive predictive value (PPV) was reported. Area under the curve (AUC) and its 95% confidence interval were calculated for each score. The ROC curve of the four scores were compared using empirical non-parametric DeLong method. Classification tree analysis was performed on the clinical variables found to be significant (*p* < 0.05) in the univariate logistic regression analysis, as a classifier for mimic stroke using entropy criterion for recursively splitting parent nodes into child nodes as the tree is grown. To prevent overfitting, the full tree is pruned back to a smaller subtree using reduced-error pruning method. Model assessment was performed using confusion matrix and cross validation approach. SAS software version 9.4 for Windows (Cary, NC: SAS Institute Inc.) was used for data analysis. Significance level was set at *p* < 0.05.

## Results

A total of 268 patients were administered IVT over the study period. However, eleven (4.1%) patients had missing clinical information and were excluded. A total of 257 patients with complete clinical information, were included for final analysis.

Two hundred and forty patients (93.3%) were diagnosed with ischemic stroke or transient ischemic attacks (TIA), while 17 (6.6%) were diagnosed as stroke mimics. Two hundred and twenty-six patients (87.9%) were diagnosed with ischemic strokes and 14 patients (5.4%) were diagnosed with TIA. Upon comparison of stroke mimics to patients with confirmed ischemic strokes/TIA, we found that stroke mimics were younger, many had history of migraine and history of psychiatric illness, and fewer had hypertension (Table 2).

**Table 2:** Clinical Characteristics of Stroke and Stroke mimics

	True Stroke (***n*** = 240)	Stroke Mimic (***n*** = 17)	Odds Ratio of Stroke mimic (95% CI)	***p***-value
Female, n (%)	81 (33.6%)	9 (52.9%)	2.19 (0.83–5.75)	0.109
Age, mean (SD)	66 (13)	57 (17)	0.96 (0.92–0.99)	0.011
Age group < 50, n (%)	36 (15%)	6 (35.3%)	3.17 (1.13–8.90)	0.041
SBP, mmHg, mean (SD)	160 (28)	161 (34)	1.00 (0.99–1.02)	0.849
DBP, mmHg, mean (SD)	91 (20)	90 (15)	1.00 (0.97–1.02)	0.801
Seizure, n (%)	2 (0.8%)	0 (0.0%)	2.72 (0.06–116)	0.706
Migraine, n (%)	2 (0.8%)	3 (17.6%)	23.0 (3.60–147)	< 0.001
Hypertension, n (%)	146 (60.8%)	6 (35.3%)	0.36 (0.13–0.99)	0.038
Hyperlipidemia, n (%)	101 (42.1%)	5 (29.4%)	0.60 (0.21–1.71)	0.305
Diabetes Mellitus, n (%)	53 (22.1%)	5 (29.4%)	1.54 (0.54–4.43)	0.485
Atrial Fibrillation, n (%)	32 (13.3%)	1 (5.9%)	0.58 (0.10–3.31)	0.375
History of Psychiatric Illness, n (%)	7 (2.9%)	3 (17.6%)	7.52 (1.79–31.5)	0.022
NIHSS on presentation, median (range)	9 (1–32)	6 (2–30)	0.98 (0.91–1.05)	0.529

DBP indicates diastolic blood pressure, *NIHSS* National Institute of Health Stroke Scale; *SBP* systolic blood pressure

**Fig. 1:**
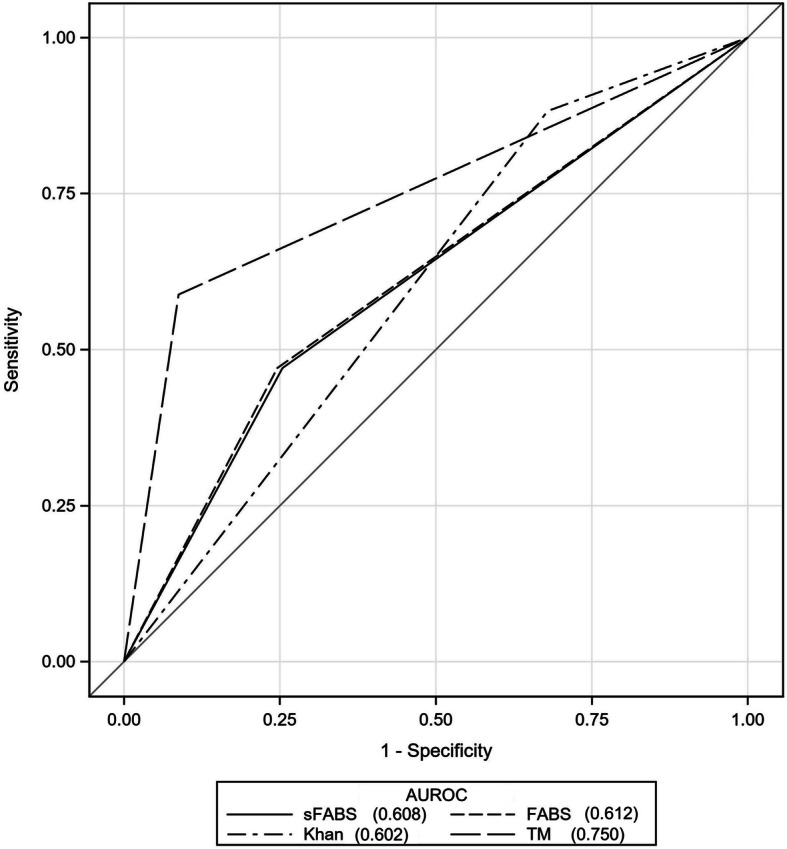
Receiver Operating Characteristics Curves for Stroke Mimic Prediction Scales. The plot of sensitivity (y-axis) vs. decremental specificity (x-axis) demonstrates TeleStroke Mimic Score with maximal area under curve. Each line represents a separate stroke mimic prediction scale and the number indicated in the legend represents the area under the receiver operating curve. AUROC indicates area under receiver operating curve; sFABS indicates simplified FABS; TM, TeleStroke Mimic Score

Among the 4 stroke mimic prediction scores, TMS had the highest discrimination for stroke mimic (AUC = 0.75, 95% CI 0.63–0.87), followed by FABS (AUC = 0.61, 95% CI 0.49–0.74), simplified FABS (AUC = 0.61, 95% CI = 0.48–0.73), and Khan Score (AUC = 0.60; 95% CI 0.52–0.69) (Fig. 1 and Table 3). The ROCs were determined based on the optimal cutoffs for each score. Additionally, the positive predictive values (PPV), negative predictive values (NPV), sensitivity and specificity for stroke mimics was calculated for each of the scores (Table 3). TMS had the highest sensitivity (91.3%), while Khan score had the highest specificity (88.2%) among the 4 scores. All 4 scores had high PPV (88.1 to 97.5%) and low NPV (4.7 to 32.3%) with Khan score having the highest PPV (97.5%). However, TeleStroke Mimic Score did not perform significantly better than the other scores.

**Table 3:** Performance Statistics for Stroke Mimic Prediction Scales

	AUROC (95% CI)	*P* value*	Cut off	Sensitivity (%) (95% CI)	Specificity (%) (95% CI)	PPV (%) (95% CI)	NPV (%) (95% CI)	LR+ (95% CI)	LR- (95% CI)
FABS	0.612 (0.487–0.738)	0.151	2	24.6 (19.3–30.5)	52.9 (27.8–77.0)	88.1 (77.8–94.7)	4.7 (2.2–8.8)	0.52 (0.3–0.91)	1.42 (0.90–2.24)
Simplified FABS	0.608 (0.483–0.734)	0.139	2	25.4 (20–31.4)	52.9 (27.8–77.0)	88.4 (78.4–94.9)	4.8 (2.2–8.9)	0.54 (0.31–0.94)	1.41 (0.89–2.22)
TeleStroke Mimic Score	0.750 (0.629–0.872)	Reference	13.6	91.3 (86.9–94.5)	58.8 (32.9–81.6)	96.9 (93.7–98.8)	32.3 (16.7–51.4)	2.22 (1.25–3.92)	0.15 (0.08–0.26)
Khan Score	0.602 (0.517–0.686)	0.081	2	32.1 (26.2–38.4)	88.2 (63.6–98.5)	97.5 (91.2–99.7)	8.4 (4.8–13.5)	2.73 (0.73–10.2)	0.77 (0.63–0.93)

AUROC indicates area under receiver operating characteristics curve; *PPV* positive predictive value; *NPV* negative predictive value; *LR+* likelihood ratio of a positive test result; *LR-* likelihood ratio of a negative test result

* *P* value of comparing paired AUC with Delong method

**Fig. 2:**
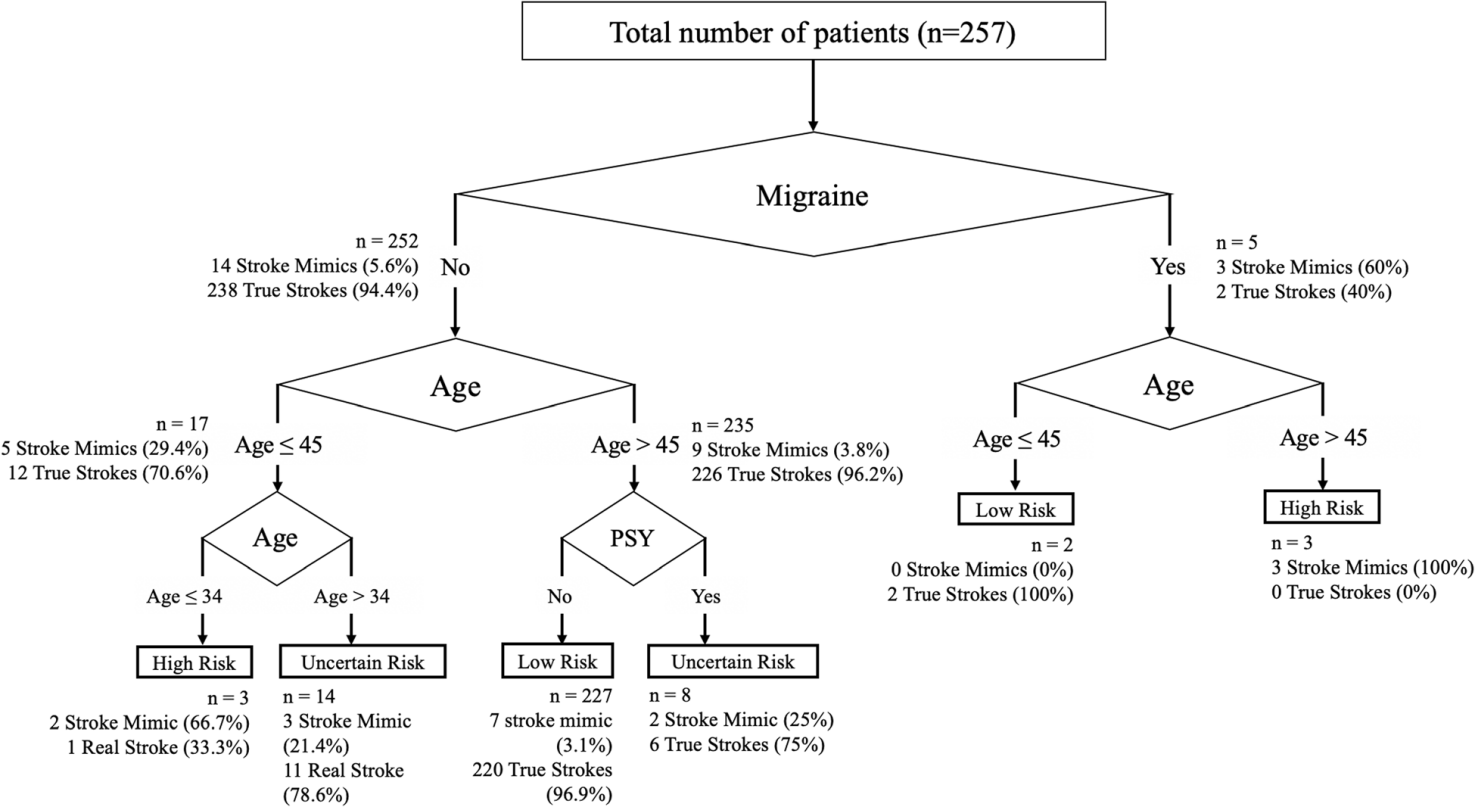
Stroke Mimic Decision Tree. The decision tree demonstrates the probability of stroke mimic or true strokes based on the clinical variables indicated. The first decision node indicates the presence of migraine, followed by age, and lastly followed by history of psychiatric illness. The number of patients at each node is indicated with the percentage of stroke mimics or true strokes indicated in brackets. A proposed risk classification of high (more than 60%), low (less than 5%) and uncertain risk of stroke mimic is indicated

A decision tree (Fig. 2) was derived from the clinical variables that were found to be significant in the univariate logistic regression analysis; namely age (as a continuous variable), presence of migraine, hypertension and history of psychiatric disorder. The decision tree suggests that stroke mimic is highly likely with the presence of migraine, and age less than 45 years. Conversely, the combination of age greater than 45 years and the absence of migraine or psychiatric history, indicated 96.9% certainty of a true stroke. The decision tree had a higher discrimination for stroke mimic (AUC = 0.80) than the 4 scales tested. Nevertheless, the classification tree has a misclassification rate of 8.6% using a 10-fold cross validation method, with 99.6% sensitivity and 47.1% specificity for stroke mimics.

**Fig. 3:**
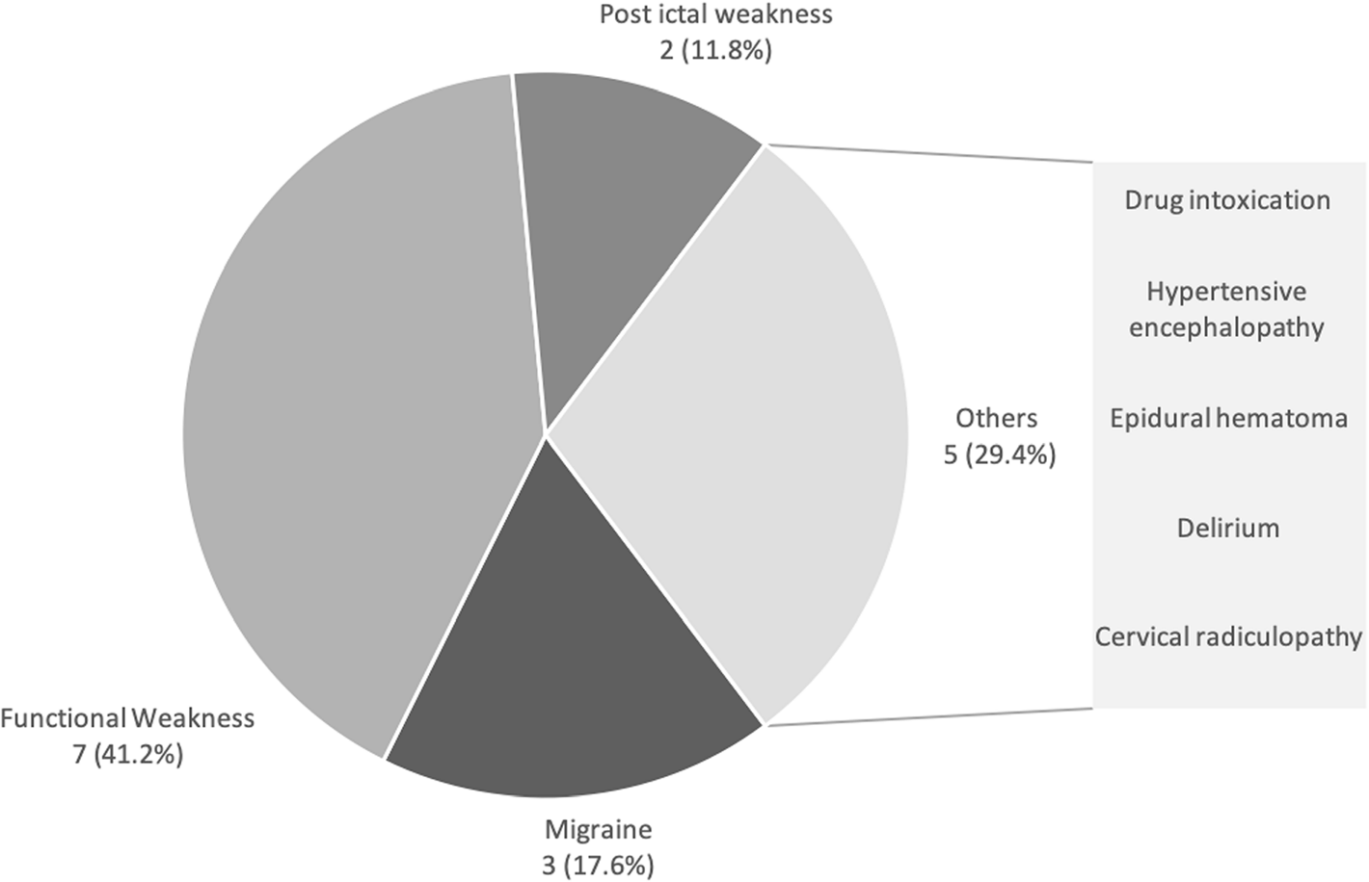
The Final Diagnosis in Stroke Mimics. The most common diagnosis made in stroke mimics was functional weakness, followed by migraine and post-ictal weakness. A single case of hemorrhagic complication in the stroke mimic was diagnosed as an epidural hematoma

The final diagnosis of the 17 stroke mimic patients included 7 functional disorders (41.2%), 3 migraine (17.6%), 2 post-ictal weakness (11.8%), 1 hypertensive encephalopathy, 1 delirium, 1 drug intoxication, 1 cervical radiculopathy and 1 epidural hematoma (Fig. 3). Among the 7 patients diagnosed with functional disorders, five had a final psychiatric disorder diagnosed either by a psychiatrist or psychologist. Two patients were diagnosed with conversion disorder, two had stress disorder and 1 patient was diagnosed with depression. Two remaining patients with functional weakness had no conclusive psychiatric diagnosis after evaluation and the cause of their neurological symptoms were unknown. Although only one of the stroke mimic (5.9%) had a hemorrhagic complication of IVT (epidural hematoma), he did unfortunately require emergent surgical treatment.

## Discussion

TMS achieved the highest discrimination performance to identify stroke mimics in our study. The AUC of TMS in our study (0.75) was similar to a previously published validation study using TMS to identify stroke mimics (AUC of 0.72) [14]. The validation study also used a telestroke cohort, similar to our current study. Nevertheless, the similar performance suggests that TMS consistently provide consistent discrimination for stroke mimics across different stroke populations. However, despite TMS having the highest AUC (0.75), the discrimination performance may be insufficient for the score to be clinically meaningful in the setting of emergency decision making for IVT, where a higher AUC may be needed. FABS and simplified FABS did not discriminate stroke mimics well in our study, as compared to the original derivation studies [9, 10]. This may be due to differing study inclusion criteria. Our study only considered patients who were administered IVT, while the derivation studies included all patients who presented with ischemic stroke symptoms. Moreover, as our study population was derived from a primary stroke centre utilising telestroke in the presence of a neurologist, some stroke mimics may have already been clinically excluded, which may potentially account for differing cohort characteristics when compared to a tertiary stroke centre. Hence, we can generalize our results only to the clinical setting of decision making for IVT, in the presence of an emergency physician, a neurologist, and a normal NCCT in a telestroke setting. Nevertheless, all of the scores were adequately rated by the study team, hence the difference of the score performance could not be completely accounted by the differences in inclusion criteria alone.

Our study was the first to externally validate Khan score [12]. Although the score had the lowest AUC in our study, it demonstrated highest specificity (88.5%) and positive predictive value (97.5%) at a cutoff of 2. The above findings suggest that Khan score may be the preferred clinical score when a high level of clinical certainty of stroke mimic is required, before any decision is made to withhold IVT. Of note, it has a low sensitivity for stroke mimics (32.1%) and poor ability to rule out stroke mimics with confidence. Conversely, the TMS has the highest sensitivity of stroke mimics (91.3%) among the four scores. Hence, it may be the score of choice if one would just like to screen the clinical possibility of stroke mimics. Given the above limitations of Khan and TMS, it may be possible for the emergency physician to use both scores for IVT decision making; the TMS as an initial screening tool and followed by the Khan score for confirmation. This will require further evaluation in a larger cohort as our current study population was not powered sufficiently to detect a statistical difference.

A clinical decision tree is an alternative method of prediction modelling compared to traditional prediction scores. Clinical decision tree has been utilised in stroke care [15] as well in other subspecialties [16, 17] to aid physicians in decision making. Similarly, a clinical decision tree may be helpful to aid emergency physicians in identifying stroke mimics by using readily available clinical information without advanced imaging. We specifically only included clinical variables, without radiological variables, in the clinical decision tree to enable it to be applicable to clinicians without advanced imaging. Because univariate analysis revealed advanced age, presence of migraine, hypertension and history of psychiatric illness to be significantly different between stroke mimic and acute ischemic stroke patients, we incorporated these variables in our derivation of the decision tree. Moreover, two of the above clinical variables (age and hypertension) were found to be replicated in the 4 tested prediction scores, suggesting that they were consistent predictors of stroke mimics.

A novel and interesting finding from our clinical decision tree analysis was that the presence of migraine was determined to be the first most important decision point in the evaluation of stroke mimics. In the absence of migraine, the next most important consideration was the age of the patient. It was surprising to find that older patients (more than 45 years) and with the presence of migraine were more likely to be stroke mimics. This is an unexpected observation, considering that all of the previous prediction scores weighed towards younger stroke mimics. The age of the 3 patients who had migraine and were above 45 years old in our database, were 48, 50 and 56 years old respectively. This suggests that although our decision tree cut off was at 45 years old, these stroke mimics with migraine were still relatively young. Our decision tree also suggests that the very young, age 34 or less, were highly likely (66.7%) to be stroke mimics. More importantly, the presence of true stroke was very high (96.9%) in patients with the absence of migraine, older than 45 years and absence of psychiatric history. This suggests that the emergency physician is relatively certain of the presence of true stroke with just 3 simple clinical factors. Overall, the performance of our decision tree had a higher AUC (0.8) than TMS. Prospective validation of the decision tree warrants a larger external cohort.

The stroke mimic rate of our study (6.6%) is considerably lower compared to other studies, that report rates as high as 26–30% [2, 18]. Although the low stroke mimic rate is likely attributable to our study design that included only patients with IVT, even in a randomised controlled trial for thrombolysis, stroke mimic rates can be as high as 16.6% [19]. Nevertheless, our finding is comparable with other studies which only included patients who had IVT and a stroke mimic rate of 3.5–4.1% [5, 20]. The majority of stroke mimics in our study were diagnosed with functional weakness, at a high rate of 41.2%. This is in contrast to seizure or migraine as the most common stroke mimic in previously published series [19, 21–23]. However, many studies have similarly found functional mimics to be the most common stroke mimic (14.5–16.7%) [24, 25], with 32% functional stroke mimics reported in a hyperacute unit [26]. This suggests that functional stroke mimics may be indeed very common across different study populations. Our study reported a single hemorrhagic complication from unwarranted IVT in a stroke mimic. Unfortunately, this particular patient required an urgent surgical evacuation of an epidural hematoma. Although hemorrhagic complication rate from IVT in stroke mimics has been reported to be low [5, 20], these complications, when they occur, may be life threatening and require urgent surgical interventions. Therefore, preventing unwarranted IVT in these numerous stroke mimics is essential and remains an unmet clinical need.

Negative neuroimaging for cerebral ischemia is common post thrombolysis. In well characterised thrombolysis cohorts, approximately 20% of post-thrombolysis patients [27, 28] had no evidence of ischemia on follow up neuroimaging. We reported 31 (12.1%) patients with negative neuroimaging in our cohort, which is lower than published literature. Although we used a combination of negative neuro-imaging findings and persistence of symptoms beyond 24 h to characterise stroke mimics in our study, some of the patients with resolution of symptoms within 24 h may be, in fact, stroke mimics rather than TIA. Nevertheless, all 14 patients (5.4%) who had complete resolution of neurological symptoms within 24 h of thrombolysis were all diagnosed to be vascular in origin by an independent neurologist not part of this study. Conversely, there could also be a possibility of the MRI-negative stroke [29] in patients with small lacunar infarctions, which were misclassified as stroke mimics. This was possible as 2 of our stroke mimics with negative imaging but had no definitive psychiatric diagnosis. This diagnostic uncertainty adds to an unmet clinical need to identify stroke patients with greater certainty.

The main limitation of our study was an under-representation of stroke mimics due to the nature of our study design. The study only included patients who were administered IVT and did not include all patients who presented to the emergency department with stroke symptoms. Because the study was performed via a telestroke network, the neurologist could have excluded stroke mimics based on other clinical factors unrecorded in our study, based on clinical experience. Therefore, there may an under-representation of stroke mimics. Additionally, due to stroke mimic patients potentially excluded, our results cannot be generalised to all patients who present with stroke-like symptoms to the emergency department. The study results can only be applicable in the clinical setting of decision making for IVT, in the presence of an emergency physician, a neurologist, a normal NCCT in a telestroke setting. However, our results may still serve as the final clinical checkpoint in IVT decision making. Our study was performed at a primary stroke centre using a telestroke system, which is different from a tertiary referral centre, with an in-house hyperacute stroke team, where stroke mimic rates may be lower. Moreover, in a centre with readily available advanced imaging, the value of the decision tree using only patient derived information alone will be lower. Regardless, in many primary stroke centres or remote hospitals all over the world, neuro imaging beyond NCCT brain may not be available. Hence, we specifically excluded any incorporation of further neuroimaging, such as CT angiogram or MRI, into the clinical decision tree. This is to focus only on clinical assessment, which is still key in clinical stroke diagnosis, and enable our decision tree to help physicians around the world without advanced imaging to ascertain the presence of stroke mimics. We did not have long term functional outcome data on the stroke mimic patients. This would have enabled us to understand and prognosticate the long-term effects of IVT in stroke mimics. We did not validate our results with an external stroke cohort, which would have strengthened our prediction model.

## Conclusion

In conclusion, TMS had the highest discrimination for stroke mimics for patients whom were administered IVT for acute ischemic stroke, in the setting of telestroke service. However, it did not perform significantly better than the other 3 scales assessed. A novel decision tree for stroke mimics was derived, which warrants an external validation in an external larger cohort.

## Data Availability

The data used and analysed during the current study are available from the corresponding author on reasonable request.

## Abbreviations

AUROC: Area under receiver operating curve
IVT: Intravenous thrombolysis
MRI: Magnetic resonance imaging
NCCT: Non-contrast computed tomography
NPV: Negative predictive value
PPV: Positive predictive value
TIA: Transient ischemic attack
TMS: TeleStroke Mimic Score
